# The Time Cost of Raising Children in Different Fertility Contexts: Evidence from France and Italy

**DOI:** 10.1007/s10680-018-9470-8

**Published:** 2018-03-09

**Authors:** Ariane Pailhé, Anne Solaz, Maria Letizia Tanturri

**Affiliations:** 1 Institut national d’études démographiques (INED), 133 bd Davout, 75020 Paris, France; 2 Department of Statistical Sciences, University of Padova, Via C. Battisti, 241, 35121 Padova, Italy

**Keywords:** Cost of children, Time use, Childcare, Paid and unpaid work, Leisure time, Gender

## Abstract

This article provides an original comparison of the time cost of children for the parental couple and for each parent in two European countries—France and Italy—that differ in terms of structural and normative constraints. Using time-use surveys carried out in 2008–2009 in Italy and in 2009–2010 in France, it investigates how Italian and French couples’ time use varies quantitatively according to the number and the age of their children. We estimate both the direct and indirect time cost of children and take into account the compression of the parents’ free time. After controlling for numerous covariates, the results corroborate the hypothesis that Italian children have a higher direct cost for couples (especially those with a large family or with preschool children), but also for mothers and fathers separately. Faced with this huge burden of childcare time, Italian women adjust by substituting housework with childcare. The presence of children reduces parents’ free time in both countries, but large families in Italy experience a higher and persistent loss of free time than in France. The gender imbalance in childcare is similar in both countries, but a more pronounced gender gap in time dedicated to domestic work is observed in Italy than in France. The loss of free time is always greater for women than for men in both countries, but in France, women’s free time is only partially affected by the number of children, contrary to Italy.

## Introduction

While most children are desired and constitute a source of happiness in modern societies, they also induce specific costs. Several studies have assessed how much children actually cost in monetary terms (De Santis 2004; Bradbury 2008; Ekert-Jaffé and Trognon 1994; Kornrich and Furstenberg 2013; Lino 2014). The mother’s loss in potential earnings because of competing parental and working times (Mincer 1963; Robinson 1987; Waldfogel 1998; Budig and England 2001; Korenman and Neumark 1992) also constitutes a significant part of the cost of children. Thus, children have a wide-ranging impact upon the allocation of time within the household (Apps and Rees 2002) and may contribute to gender inequalities. However, little is known about the cost of children in terms of time, how much it differs for mothers and fathers and how much it varies according to the structural and institutional context. This article provides an original comparison of the time cost of children for the parental couple and for each parent in two European countries—France and Italy—that differ in terms of structural and normative constraints.

Studies that include the time costs of children often concentrate only on the mere input of parental time devoted to children (basically, time allocated to childcare and activities for children), which is usually defined as the *direct time cost of children* (Sayer et al. 2004; Pailhé and Solaz 2008). They show that parents now spend more time with their children than they did in previous decades (Bianchi et al. 2000; Sayer et al. 2004 for the USA, Gauthier et al. 2006 for selected industrialized countries). This may be linked to the fact that social expectations with regard to parenting have increased (Coltrane 2007; Craig and Mullan 2010) along with parental expectations relative to their children’s human capital development (Becker and Tomes 1986). However, children do not only affect time spent on childcare; they are also likely to affect time spent on all other activities, including domestic tasks (Apps and Rees 2002; Gustafsson and Kjulin 1994), by changing the overall time allocation.

The possibility of freely managing one’s own time is considered to be more and more important for individual fulfilment (Lesthaeghe 1995; van de Kaa 1987). However, given that daily time is limited, pure leisure time and time spent on personal care (e.g. sleeping, bathing) may also be affected because child-related activities are time-consuming and in competition with other sources of satisfaction (e.g. travelling, going to the cinema). Following the same principle that money is diverted into child-specific goods instead of adult goods when a child is born into a household, a parent’s time is redirected from their leisure and personal time towards the children’s specific needs (Craig and Bittman 2008; Ekert-Jaffé and Grossbard 2015). The quantity of remaining free time can be interpreted as an indicator of parents’ well-being (Cross 2005; Zabriskie and McCormick 2003), as its excessive compression might cause dissatisfaction or even psychological distress. This reduction in parental time for personal needs is referred to in this paper as the *indirect time cost* of children.

Since the anticipated cost of children might be an important factor influencing parental fertility decisions (Bradbury 2008), studying the time cost of children is a key issue for demographers. Italy and France represent particularly interesting cases in this respect (Régnier-Loilier and Vignoli 2011). Fertility in France remains close to the replacement level, while Italy has a completed cohort fertility of 1.5 (Table 5). In both countries, the two-child family is the dominant norm, but childless and one-child families are more frequent in Italy than in France.

These two neighbouring countries differ in several domains that influence time use, in terms of structural, institutional and normative settings. First, the participation of women in the labour market is substantially higher in France than in Italy. France is also a more family–friendly environment than Italy (Thevenon 2016; Tanturri 2016). These two countries, however, share similar normative determinants of the time cost of children, e.g. Catholic values, Latin cultural heritages and asymmetric gender roles. Attitudes and values are nevertheless more conservative in Italy than in France, and familialism is much more deeply rooted (Dalla Zuanna 2001; Dalla Zuanna and Micheli 2004).

This paper provides a cross-country comparison of the time cost of children and its determinants. It investigates how Italian and French couples’ time use varies quantitatively according to family structure, i.e. number and age of children. We exploit Italian and French Time-Use Surveys, carried out, respectively, in 2008–2009 and 2009–2010. We estimate the *direct* and *indirect time cost of children,* and we take into account the “net adjustment” of parental time devoted to paid and unpaid work by looking at the compression of the parents’ time for pure leisure and personal care, defined as *free time*. Thanks to time-use data that provide diaries for several individuals per household, we measure and compare the total workload at the couple level in different types of family between the two countries. As these costs associated with children are usually distributed unequally between partners, we also analyse the adjustment of the various time components within the couple and for each parent. This paper adopts a descriptive approach rather than a causal one, as the latter would require panel time-use data that are not available for the selected countries.

## Background

From a theoretical point of view, three main groups of determinants underlie individual time allocation, i.e. structural, institutional and normative determinants (Gauthier and DeGusti 2012). In this framework of analysis, France and Italy reveal major differences and a few commonalities.

### Structural Determinants

A first determinant of the time cost of children is related to *time availability*. From this perspective, women’s and men’s domestic time depends on time spent in paid work and on work schedules (Bianchi et al. 2000). This allocation of time to housework is related to differences in spouses’ comparative advantage in the labour market (Becker 1991) and to spouses’ relative resources, such as level of education or income (Brines 1994). It also depends on employment opportunities, the tax system and the availability and cost of childcare.

Women’s labour market participation differs between France and Italy (Table 1). It is higher in France, but the average working hours are longer in Italy. Long work schedules and scarce part-time employment opportunities could be seen as obstacles for Italian women seeking to reconcile their work and family, such that many of them are obliged to either give up their career as soon as they have family responsibilities or to limit the number of children they have. The dominant family model in France is that of two full-time earners, and the societal acceptance of working mothers is high among both individuals and employers, even for mothers with young children; 59% of French mothers with children aged under 3 are in employment. In Italy, maternal employment is rather low (Del Boca et al. 2005), does not vary much by the age of the youngest child and significantly decreases with the number of children. It is common for working women to exit the labour market after having a child: one in five pregnant working women are no longer in the labour market when the child is around 2 years old, and this proportion has increased in recent years (Istat 2014); 53% of Italian mothers of children aged 3–5 are in work, compared to 74% of their French counterparts.

**Table 1:** Maternal employment rates by age of youngest child and by number of children, 2009 (%)

	Age of youngest child			Number of children		
	< 3 years	3–5 years	6–14 years	1 child	2 children	3 children or more
France	59.3	73.7	79.7	75.8	73.6	52.3
Italy	52.2	53.2	55.5	57.4	51.2	37.7

*Source*: OECD family database http://www.oecd.org/els/family/oecdfamilydatabase.htm

### Institutional Determinants

In the literature, policies and institutional determinants are commonly seen as important correlates of time use (Gershuny and Sullivan 2003; Sullivan et al. 2009). The time devoted to childcare—and thus the time cost of children—is strongly influenced by the opportunities for outsourcing housework and childcare, the organization of time schedules, and policies that are more generally family–friendly. France and Italy present remarkable differences in terms of public spending for families, family policies and childcare services (Table 2). In France, family policies have a long tradition (Thevenon 2016) and public expenditures on families are among the highest of OECD countries (3.98% of GDP in 2009, the OECD average being 2.6%). The family policy package combines benefits designed to reduce the cost of children for families, parental leave and subsidized day care (day-care centres, subsidized registered childminders and nursery schools), where a child can be placed from the age of 2½ months to 3 years. Since 2004, specific tax deductions have been granted for private firms that actively provide childcare or develop measures to help their employees improve their work–family balance (Toulemon et al. 2008). The availability of different types of low-cost means-tested childcare with long opening hours (up to 11 h a day for day-care centres) supports maternal employment. The system of joint taxation of couples who are married has limited disincentive effects on female employment (Carbonnier 2014). Concerning housework, French households who employ a domestic helper can deduct half of the cost from their income tax (Marbot and Roy 2011).

**Table 2:** Enrolment rates of children in formal and informal childcare (%) in 2008 and average hours of attendance per week in formal care in 2010, by child’s age

	Formal care or early education services		Informal childcare arrangements^a^			Average hours of attendance per week in formal care
	0–2 years	3–5 years	0–2 years	3–5 years	6–12 years	0–2 years
France	42.0	99.9	17.7	19.6	13.6	31
Italy	29.2	97.4	31.5	37.0	29.2	29

*Source*: OECD family database http://www.oecd.org/els/family/oecdfamilydatabase.htm.

^a^2007 for France

In Italy, on the other hand, public support for families is very limited (e.g. total expenditures represent only 1.58% of GDP), and there is no clear and consistent system of family–friendly policies (Naldini and Saraceno 2008; Tanturri 2016). Furthermore, there is no universal child benefit in Italy and the tax system takes children into account only minimally (Naldini and Saraceno 2008). Reliance on relatives to provide social support is much greater in Italy than in France. The work-life balance is certainly more difficult in Italy, due to the limited supply of public childcare for children younger than three, specifically in terms of availability, number of hours of care provision and cost (Istat 2014; Tanturri 2016). Private childcare facilities, for their part, are limited and expensive.

Cross-country differences are less pronounced for children aged three and over, since the age at entry into the formal school system is 3 years for both countries. However, the hours covered by the service are different: French school hours are from 8:30 to 16:30 for all children aged 3–12, while in Italy similar hours are generally available only for children aged 3–5. Only one-third of Italian primary school pupils (6–11 years old) can stay in school all day long. This arrangement is available only on request, and the number of places is limited; the majority of children attend school for just 24 or 27 h per week. For children aged 10–13, the Italian standard school hours are 8:00–13:00, 6 days a week, without any school meal service. In addition, care facilities before and after school hours are much more widely available in France, while in Italy the family is expected to provide for all extra-curricular activities.

### Normative Determinants

The time devoted by men and women to children and housework is strongly influenced by norms, especially those related to gender roles, domestic standards and good parenting (South and Spitze 1994). First, the time devoted to childcare and housework depends on expectations and attitudes about housework (Robinson and Milkie 1998). France and Italy are quite similar in that respect; for instance, they share lifestyles that value family time and the importance of food—especially family meals—in daily life (Davidson and Gauthier 2010). Second, according to the gender perspective, men and women conform to their own gender roles through the amount and type of housework they perform (Ferree 1990; South and Spitze 1994). Italy and France are not so dissimilar in that respect, as their Catholic religious values and Latin cultural heritage favour asymmetric gender roles. For instance, they have similar rankings on the UN gender inequality index (rank 20 for France and 26 for Italy in 2013). Attitudes and values are nevertheless more conservative in Italy than in France. France is more secularized, and gender roles are shaped in less traditional ways than in Italy (Anxo et al. 2011). Indeed, Italian women are more often homemakers, spend more time on unpaid household work and usually perform a higher share of domestic and childcare tasks (Mencarini et al. 2017; Anxo et al. 2011; Mencarini and Tanturri 2004; Gauthier and DeGusti 2012). The public view of working mothers in Italy is still particularly negative: European Social Survey data (2008) indicate that three quarters of Italians versus 41% of the French think that a preschool child suffers to some extent if his/her mother works. These traditional norms are supported by public policies and structural factors. In France, until 2015, there were no explicit policy measures to encourage fathers’ involvement with their children apart from the 10-day statutory paternity leave introduced in 2002. Italian working fathers (in the private sector) have only been entitled to a 1-day paternity leave since 2012. Parental leave has been available to men since 2000, but uptake is still very limited (Tanturri 2016). At the same time, work regulations and schedules often prevent fathers from contributing to childrearing (Ruspini and Tanturri 2015) and this, together with the paucity of public childcare, reinforces the gender imbalance. Strong family ties (Reher 1998), familialism (Livi Bacci 2001, Dalla Zuanna and Micheli 2004) and an almost exclusive reliance on family members for material and emotional support increase women’s responsibilities within the family in Italy.

### Research Hypotheses

Given the cross-country differences in the structural, institutional and normative determinants of child time costs, we expect childrearing to be more parental time intensive in Italy than in France. The provision of childcare facilities in France relaxes the parents’ time constraints and alleviates the parental burden in terms of childcare. *We thus expect a lower direct time cost of children in France than in Italy* (H1). As two of the most salient differences between Italy and France concern the provision of childcare services for children under three and the school attendance time for older children, we thus expect the cross-country differences in child time costs to be higher in terms of childcare than in terms of additional housework and reduction in free time.

The cross-country differences in housework time are related to normative determinants and to structural constraints such as the opportunities to outsource housework and childcare, which are greater in France than in Italy. *More balanced gender roles and lower normative constraints regarding housework may be associated with a more favourable institutional background and thus reduce the time costs in terms of additional housework for French women compared with their Italian peers* (H2).

Regarding *free time, we expect the Italian parents to have a much higher compression of time devoted to relaxation, personal care and leisure than their French peers, as the time costs in terms of childcare and housework in Italy are not counterbalanced by lower labour market participation* (H3). Finally, given the unequal gender roles in both countries, we expect the cost of children to be higher for women than for men. However, *the gender gap is expected to be more pronounced in Italy than in France* (H4), as it is reinforced by pervasive familistic values and structural constraints.

## Data and Methods

### Data

Time-use survey data provide a unique source for measuring the cost of children in terms of parental time devoted to childcare. The diary technique (whereby individuals report their time use during a period of 24 h) provides extremely detailed information on the activities performed throughout a certain day (or over several days). The diary data are based on a grid of 10-min intervals of time, with a description of the main activity carried out by the respondent, the second (or concurrent) activity, their location and the presence of other persons. Aside from the diary, all the datasets contain detailed information on the background and socio-economic situation of individuals and households.

The French Time-Use Survey used for this study was conducted in 2009–2010 by the French National Institute of Statistics and Economic Studies (INSEE). A total of 17,383 respondents were asked to record their time use. In Italy, the time-use survey used was carried out as part of the Multipurpose Surveys Project conducted by the National Institute of Statistics (ISTAT) in 2008–2009 on a sample of 44,606 individuals. The diary was filled in for all household members aged 3 years or over in Italy, while in France it covered a maximum of two persons aged 11 or more; only one person was randomly chosen from among all household members over age 11, and if this person had a co-resident partner, he or she was also surveyed.

The diary days in both countries were randomly distributed across days of the week and throughout the year. One daily diary was filled in on either on a weekday or a weekend day in the Italian survey, while respondents in the French survey filled in either one or two diaries (one on the weekend and one on a weekday). In both countries, both spouses filled in the diaries for the same day.

The sample is composed of adult couples (2578 couples for 3781 diaries in France and 3597 couples and diaries in Italy), either married or cohabiting, in which both partners are aged 20–54 and are either childless or have at least one child under 13. Households with adults in addition to the couple were excluded from the sub-sample, as well as complex families, in order to avoid the confounding effect of other adults who are able to provide childcare or domestic tasks but might also require additional care. In the study sample, 26.9% of the couples in France and 21.4% in Italy were childless, while the others had at least one child under 13 years old (Table 3).

**Table 3:** The sub-sample in Italy and France by family composition (absolute frequencies and column percentages)

	France		Italy	
	*N*	%	*N*	%
Childless	1161	30.7	768	21.4
1 child, aged				
0–2	366	9.7	364	10.1
3–5	225	6.0	276	7.7
6–12	207	5.5	360	10
2 children, the youngest aged:				
0–2	363	9.6	372	10.3
3–5	340	9.0	347	9.7
6–12	500	13.2	708	19.7
3 + children, the youngest aged:				
0–2	190	5.0	119	3.3
3–5	182	4.8	98	2.7
6–12	247	6.5	185	5.1
Total	3781	100	3597	100

*Source*: Italian Time-Use Survey 2008–2009 and French Time-Use Survey 2009–2010

### Methods

The methodology is based largely on a seminal paper on Australia by Craig and Bittman (2008). The time cost of children is assessed by comparing the daily workload in time for couples and individuals with and without children. Only adults’ time is analysed in both countries.

The analysis is run in three steps. First, the cost of children is measured in terms of the time devoted directly to *childcare*. This time is defined on the basis of activities devoted directly and exclusively to children in the household, i.e. physical care (feeding, washing, medical care, etc.), interactive childcare (conversations, reading, playing games at home or outdoors, artistic activities, sports and excursions), homework supervision and transportation of children. This definition measures active childcare and is thus lower than total time spent in the presence of children (see Tables 6 and 7 for alternative measures). Second, the time cost of children is estimated by taking into account the amount of *housework*, under the hypothesis that a child also engenders an increase in the time dedicated to other domestic tasks, such as cooking meals and cleaning or tidying up (Craig 2007; Craig and Mullan 2010). *Housework* also includes all other domestic tasks such as doing the washing, shopping, paying bills, household management, small repairs, care of family members other than children and transportation related to these activities. Finally, the *indirect child time* cost is measured in terms of *foregone free time*, i.e. the time dedicated to personal care (e.g. eating, sleeping and bathing) and to pure leisure (e.g. sport, cultural activities, walks and conversations), that is reduced by the presence of children. The remaining time is working time.

We refer only to the primary activity recorded in the diary, which may appear as a restrictive definition since parents—especially mothers—very often care for their children, while they are performing some other “main” activities (Craig 2007; Folbre et al. 2005). This choice is guided by both practical and theoretical considerations. One practical reason for this decision is that secondary activities are not recorded exhaustively and do not always seem to be done in the same way in both the Italian and French surveys.^1^ Using such partial information to estimate child costs may create huge biases. From a theoretical point of view, it is also debatable whether the secondary or concurrent activity would lead to a more realistic assessment of the true time costs. Our argument is that it is most important to define whether the type of primary activity (be it housework or leisure, for instance) is costly or not. If the primary activity is housework, passive care is indirectly included in the time cost of the child, since doing domestic work is considered to be part of some incremental time cost. If it is leisure, the presence of children might have an ambiguous effect, since it may either disrupt the leisure period or conversely be appreciated by the parents, who therefore would not count it as a time cost.

In order to test our first and third hypotheses, we measure the couple’s total workload. These times are computed at the couple level, i.e. summing the time spent by each spouse (whether spent jointly or not). In order to test our second and fourth hypotheses, we observe the time spent by women and men separately.

One main problem when analysing time-use data in regression analyses is that some individuals may not spend any time on some activities on the day of the interview. If this is true, the dependent variable defined by the time spent on a certain activity is not normally distributed (for observations with a value equal to zero), and simple linear regression analysis may bias the results. A common solution used in some previous studies is to use a Tobit regression. However, its advantages in comparison with ordinary least squares (OLS) have been recently challenged (Domínguez-Folgueras 2012; Foster and Kalenkoski 2013; Stewart 2009; Neilson and Stanfors 2014; Pacholok and Gauthier 2010), especially when the zeros reflect real behaviour rather than under-reporting. Our choice is in favour of linear estimations, since we believe that 0 min performing an activity is attributable to real behaviour rather than censoring. Furthermore, the results from linear estimations are more easily and directly interpretable in hours and fraction of hours.

In order to take into account interrelations between different types of time use, we jointly estimate time devoted to *childcare*, to *housework* and to *free time* (Kimmel and Connelly 2007; Kalenkoski et al. 2005, 2007). This system of equations is first estimated for the couples (3 equations) allowing for correlations among the error terms of each equation. Then, another system is estimated jointly for men and women (6 equations) to capture the possibility that unobserved characteristics might simultaneously affect the time allocation of individuals as well as time sharing within the couples.

The time cost of children is assessed by taking the daily workload of childless couples and comparing it with couples who have different numbers of children (0, 1, 2, 3 or more). We also consider the age of the youngest—plausibly the most demanding—child (aged 0–2, 3–5, or in primary school). In this way, we can calculate the marginal differences in workload (or incremental time) associated with each extra child, assuming that the observables introduced (see below) make the family situations comparable. In Table 3, the absolute and relative frequencies for each type of family are shown.

Since time budgets provide only cross-sectional and not longitudinal data, the results must be interpreted with caution. If being a parent is linked to the time allocation or to the gender division of housework that existed prior to parenthood, our findings may be biased (Kalenkoski et al. 2007). Indeed, if those who prefer large families spend more time doing housework even before having children, the arrival of a child does not alter their time use very much. In the absence of individual information over time, we must be aware that our cross-sectional design implicitly assumes that, without children, parents would have used their time in the same way as those who are currently childless, or that parents with three children used their time as parents with two children when they had only two.

We introduced a large set of control variables, however. Conditional upon these observable characteristics, childless couples and parents are comparable.^2^ We include the age of each partner (in three age groups: 25–34, 35–44 and 45–54 years) and their marital status. We also include precise indicators of the relative educational attainment of both partners by placing them in seven categories: (1) both partners have high level of education, i.e. a university degree; (2) both partners have an intermediate level of education, i.e. a secondary school certificate; (3) both partners have low level of education, i.e. less than a secondary school certificate; (4) man with high and woman with lower education; (5) man with intermediate and woman with low education; (6) man with intermediate and woman with high education; (7) man with low and woman with high education. When regressions are run separately on men’s and women’s time use, we consider each partner’s level of education separately and not in combination. We also add dummies describing the couple’s labour supply: (1) both partners work full time; (2) man works full time, and woman part time; (3) man works full time, and woman is homemaker; (4) a residual category includes men not working full time. To control for economic conditions, dummies related to household income are also included in the model. For France, quartiles of income per consumption unit are used. Since household income is not available in the Italian data set, a covariate describing the household’s self-reported economic resources is added. Economic resources can be estimated as (1) more than sufficient, (2) just sufficient or (3) insufficient. As labour market situation and income might be potentially endogenous, an alternative specification without couple’s labour supply variables was also estimated to check that our results are not driven by the introduction of these variables.^3^ Other controls include a dummy for the households that outsource part of their childcare and domestic tasks to a paid helper. Three indicator variables for the days of the week are controlled for in the model: (1) weekday; (2) Saturday; and (3) Sunday. Finally, indicator variables on the geographical area are added to specify the size of the locality: (1) small (fewer than 50,000 inhabitants), (2) medium (more than 50,000 inhabitants, but not a metropolitan area) and (3) a large city (metropolitan area and suburbs). The model for Italy introduces three dummies for the macro region (i.e. North, Centre and South), while a dummy variable for France indicates whether the respondent lives in the Parisian area or not. “Appendix” Table 5 provides a description of all the covariates.

Finally, we performed a model on the pooled data of the two countries (limited to strictly comparable variables between countries), including interactions between the country and family structure variables (“Appendix” Table 10) in order to estimate the additional time spent in one country relative to the other.

## Results

Couples devote 2.4 h a day on average to childcare in Italy and 2 h in France (Table 4). Both Italian men and women devote more time to their children than their French peers. However, fathers in both countries assume the same share of total childcare: approximately one-third. Cross-country differences are higher in terms of time spent on housework. Italian women spend 1.8 h more on housework than French women. In contrast, men spend more time on housework in France than in Italy, both in absolute and in relative terms: Italian men account for 24% of the time that couples devote to housework and French men 38%. Moreover, the latter have less free time than in Italy, while French women enjoy more free time than their Italian peers. Thus, a similar level of free time for the couple in both countries is shared differently between partners in France and Italy.

**Table 4:** Mean value of childcare, housework, leisure and paid work times in Italy and France (hours per week)

	Italy						France					
	Total		Parents		Childless people		Total		Parents		Childless people	
	Mean	SD	Mean	SD	Mean	SD	Mean	SD	Mean	SD	Mean	SD
Childcare												
Couples	2.4	2.6	3.0	2.6	0	0	2.0	2.4	2.8	2.4	0.2	0.7
Women	1.6	1.9	2.1	1.9	0	0	1.3	1.7	1.9	1.8	0.1	0.5
Men	0.8	1.2	1.0	1.3	0	0	0.7	1.1	0.9	1.3	0.1	0.4
Housework												
Couples	6.5	3.6	6.5	3.5	6.3	4.0	5.1	3.5	5.2	3.4	4.9	3.7
Women	4.8	2.7	5.0	2.6	4.3	2.7	3.1	2.2	3.2	2.1	2.9	2.3
Men	1.6	2	1.5	2	1.9	2.2	2.0	2.2	2.0	2.2	2.0	2.2
Paid work												
Couples	6.9	6.9	6.9	6.7	7.2	7.5	8.1	7.5	7.9	7.3	8.4	7.9
Women	2.0	3.5	1.8	3.3	2.7	4.0	3.2	4.1	3.0	4.0	3.6	4.3
Men	4.9	4.9	5.0	4.9	4.5	4.8	4.9	4.8	4.9	4.8	4.8	4.7
Free time												
Couples	32.1	7.0	31.5	6.8	34.5	7.1	32.8	6.8	32.0	6.5	34.6	7.1
Women	15.5	3.5	15.1	3.3	16.9	3.6	16.4	3.6	15.9	3.4	17.4	3.7
Men	16.7	4.3	16.4	4.2	17.6	4.3	16.4	4.1	16.1	4.1	17.2	4.2
N	3597		2829		768		3781		2620		1161	

*Source*: Italian Time-Use Survey 2008–2009 and French Time-Use Survey 2009–2010; authors’ computations

*SD* Standard deviation

**Fig. 1:**
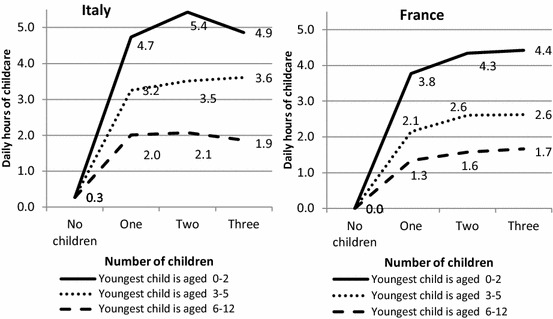
Predicted daily hours of childcare performed by couples in Italy and France according to the number of children and age of the youngest child, net of other confounders (*on weekdays for a reference couple*)

**Fig. 2:**
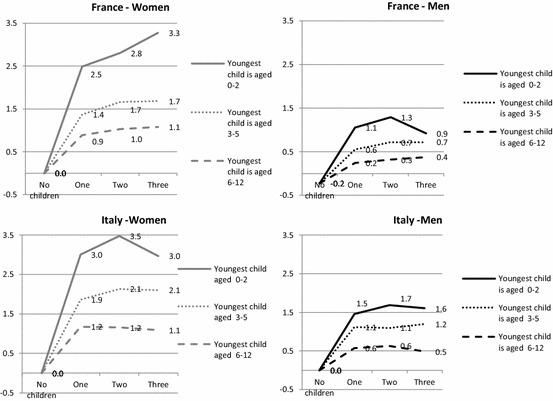
Predicted daily hours of childcare performed by men and women in Italy and France according to the number of children and age of the youngest child, net of other confounders (*on weekdays for a reference woman or man*)

**Fig. 3:**
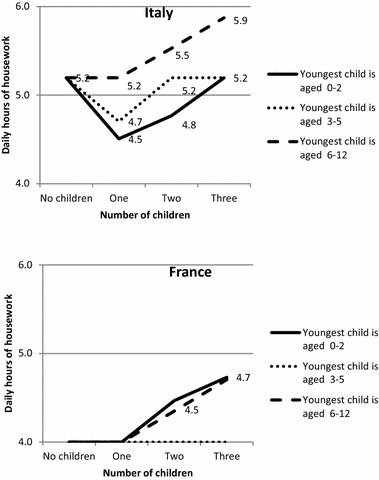
Predicted daily hours of housework performed by couples in Italy and France according to the number of children and age of the youngest child, net of other confounders (*on weekdays for a reference couple*)

**Fig. 4:**
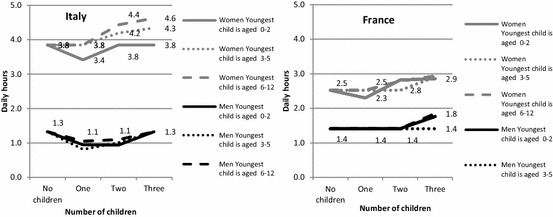
Predicted daily hours of housework performed by men and women in Italy and France according to the number of children and age of the youngest child, net of other confounders (*on weekdays for a reference woman or man*)

To facilitate cross-country comparison, we present the results in Figs. 1, 2, 3 and 4, showing how the predicted daily hours of work (on the vertical axis) evolve with the number (horizontal axis) and age of children (0–2, 3–5, 6–12), all other things being equal.^4^ The parameters of the full regressions are shown in “Appendix” Tables 8, 9 and 10, together with their standard errors.^5^

### The Direct Time Cost of Children: Parental Time in Childcare

Couples with one child under three spend a considerable amount of time on childcare, all other things being equal: almost 5 h a day in Italy and almost four in France (Fig. 1). A child aged 3–5 requires less care, as most children at that age go to nursery school in both countries: their parents devote 3.2 h per day to childcare in Italy and 2.1 in France. The childcare time drops to 2 daily hours after age 5 in Italy and to 1.3 h in France. Whatever the number of children in the family, having at least one preschool child always dramatically increases the total time spent on childcare by parents. This huge effect of age might be partly driven by the decision to restrict childcare to primary activities in our definition.^6^

The couple’s childcare time decreases substantially by the age of the youngest child, but not by the number of children. In Italy, having at least one preschool child plus one other child increases childcare costs by up to 5.4 h a day, but the profile of child costs is relatively flat when the number of children increases and the youngest child is older. One possible reason behind this pattern is the economies of scale achieved when several children are looked after together, which allows parents to save time. Older siblings might actively take care of their younger brothers and sisters, so that their parents spend less time overall on active childcare. A selection effect might also operate: parents of large families may have more offspring because their first experience of childrearing was less time-consuming, they are more efficient at performing childcare activities or, finally, they give lesser importance to investment in child quality, in line with the well-known trade-off between quality and quantity.

The pooled model (“Appendix” Table 10) shows that French couples always spend less time on childcare than Italian couples, other sociodemographic characteristics being equal. The largest difference between France and Italy is in the additional cost of having a preschool child or a young child in Italy. *Ceteris paribus*, Italian parents of one or two children with a child below 6 spend almost 1 h more per day than French parents and around a half an hour more when the last child is older. The predicted differences could be explained by the lesser difficulty in outsourcing childcare as well as longer school hours in France (H1). The lack of external childcare arrangements in Italy means that parents are the main providers of childcare. However, we cannot exclude the possibility that Italian parents spend more time with their children just because they want to, which follows the social norm that family members are children’s best caregivers.

This household-level child cost is evenly distributed between mothers and fathers (Fig. 2). The mothers of one preschool child spend between 3.0 and 3.5 h on childcare per day in Italy and around 2.5–3.3 h in France. Men are substantially less involved in both countries and spend about half as much time as the mother in taking care of their children. Italian fathers of one preschool child spend around 1.5 h in childcare per day, around 25% more than their French counterparts.

The results for couples corroborate the hypothesis that Italian children consume more parental time than French children (H1). At the same time, women are much more involved in childcare activities than men in both countries. However, both Italian mothers and fathers spend more time on childcare than their French counterparts (“Appendix” Table 10); thus, the gender gap is surprisingly similar in both countries and the hypothesis that the gender gap is wider in Italy (H4) therefore appears to be unverified for the direct cost of children.

### The Domestic Workload Due to Children

Childless couples perform a remarkable amount of housework in both countries, but with important differences: 5.2 h daily for both partners in Italy, i.e. over 1 h more than in France (Fig. 3). Evidently, this difference reflects different expectations and norms about household duties and standards in the countries under focus, even in the absence of children. Though paid domestic help is controlled for in the model—but probably not always reported in case of the use of undeclared workers—these differences may also be related to the tax rebates available in France for hiring a domestic helper.

In contrast to what is observed for childcare, housework time varies only slightly with family composition, and not always in the expected direction. In France, it increases for those having two or more children. In Italy, the domestic workload is even lower for couples having children under six than for childless couples. Conversely, it is higher among couples having at least two children of whom the youngest is older than five. Italian parents with only one child or at least one preschool child abandon some domestic tasks (or do them simultaneously with other tasks) in order to spend more time with their children. This substitution effect is confirmed by the negative and significant sign of the correlation between the errors of the equations between domestic time and childcare times in Italy. This is not the case in France.

The differences between the two countries in terms of incremental housework time in the presence of children are relatively smaller than those observed for childcare (“Appendix” Table 10). As Italian couples already spend a lot of time on housework when they are childless, having children involves few quantitative changes for them and even leads to a reduction in housework time in some cases, whereas the time French parents spend on housework remains at the same level or increases.

The gender difference in time devoted to housework by childless couples is much larger in Italy than in France (respectively, 2.3 and 1.1 h a day, Fig. 4), reflecting a gender imbalance that is not linked to the presence of children. As for childcare, the domestic workload is borne mainly by women in both countries (Fig. 4), but, in this case, the gender gap is much wider in Italy than in France. Hence, while Italian women perform between 74 and 81% of domestic tasks, depending on family composition, French women do between 61 and 67%. Italian women also have a much higher domestic workload in absolute terms: they spend no less than 3.4 h a day on housework, whatever the family composition, while in no case do their French peers perform more than 2.9 h. Conversely, Italian men do less housework than French men.

The variation by family composition also differs between countries. In France, the time devoted to housework by household composition is quite stable for both men and women, except the slight increase observed for large families. In Italy, the time devoted to housework by men decreases when they have one or two children. Therefore, for Italy, the substitution effect—that we noticed for the couples—applies only to fathers, who reduce the limited time they devote to housework to care for their children, and by the mothers of one preschool child. For women, the incremental cost is considerable for larger families with older children in Italy, but, as with men, the child’s time cost in terms of housework is lower than for childcare time.

The cost of children is lower for French women than for Italian women in terms of additional housework. This evidence seems to corroborate our hypothesis (H2), but it is true primarily for women with 2 or more children over 3 (around 30 min, see “Appendix” Table 10). In both countries, women with a preschool child have a similar reduction in time spent on housework. It also suggests that older children might be asked more often to help with domestic tasks in France, or from an earlier age, than in Italy, as has been shown for young adults (Mencarini et al. 2017). In spite of their greater involvement in childcare, Italian men spend less time on housework than their French peers, whatever the family composition.

### Forgone Free Time

The amount of free time is always lower in Italy than in France, whatever the family composition. Most French couples devote less than 27 h a day (out of 48 h) to free time, while in Italy only childless couples have more than 27 h of free time. This might be due to the higher housework workload and to longer working hours in Italy than France.

**Fig. 5:**
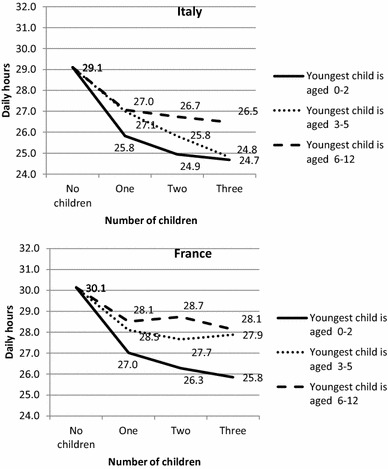
Predicted daily hours of free time performed by couples in Italy and France according to the number of children and age of the youngest child, net of other confounders (*on weekdays for a reference couple*)

Not surprisingly, in both countries, having children reduces time for leisure and personal care by at least 2 h per day, especially when the children are younger and the family is large (Fig. 5 and Table 8). Hence, compared to childless couples, the presence of one child aged 0–3 is associated with a reduction in free time of 3.1 h per day for a French couple and 3.3 h for an Italian one. The daily loss of free time is particularly high for couples with at least three children of whom the youngest is under 3 years old: 4.3 h in France and 4.4 h in Italy, respectively. These differences between childless couples and parents of children under 5—in terms of free time—are lower than the observed increment of childcare. Therefore, this means that couples with young children also adjust their paid labour supply to preserve free time. Nevertheless, this decrease in working hours does not entirely offset the increase in total unpaid work, and parents of young children have much less time than childless people to spend as they wish and to rest.

**Fig. 6:**
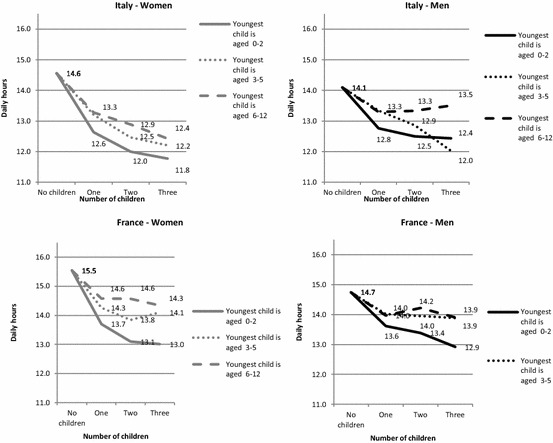
Predicted daily hours of free time performed by men and women in Italy and France according to the number of children and age of the youngest child, net of other confounders (*on weekdays for a reference woman or man*)

Forgone leisure and time for personal care is higher in both countries for women than for men (Fig. 6). For mothers, the reduction in free time—compared to childless women—varies between 1 h and 2.5 h per day in France and between 1.3 h and 2.8 h in Italy. Although men spend more time in paid work, these figures are lower for men, at between 0.5 and 1.8 in France and 0.6 and 2.1 in Italy. We can therefore conclude that this indirect cost of children is higher for women than for men in both countries, and it is higher in Italy than in France.

**Fig. 7:**
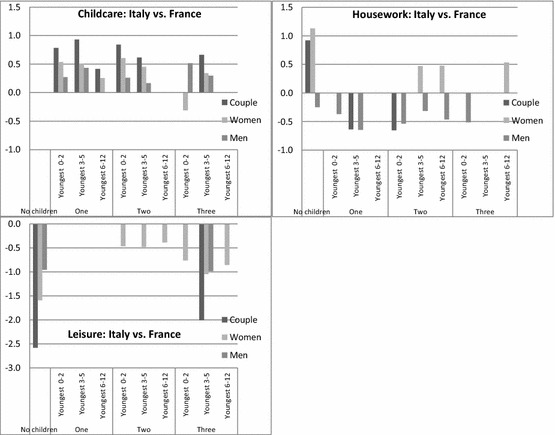
Results of the interaction of country and family situation (pooled model). *Note* Simultaneous equations model on childcare, housework and free time, performed on pooled data between countries with country dummy, controlled for education level, age, activity status, marital status, family composition, domestic help, town size and day of interview. The interactions between country and family structure are represented

H3 appears to be verified as the time costs of children in terms of forgone leisure and personal time are always greater in Italy:^7^ this means that the higher time cost in terms of childcare and housework is never counterbalanced by the lower labour market participation. This occurs despite the fact that Italian childless couples already have less free time than their French peers: indeed, a French childless couple has 2 h more free time a day than an Italian one (Fig. 7). For one-child families, the cost of children in terms of forgone free time is very similar between countries. In contrast, large families have a higher cost in terms of lost leisure in Italy than in France. Even more importantly, Italian mothers of two or three children are much less likely to participate in the labour market than their French peers, yet their loss of free time is greater (H4).

## Conclusions

This paper compares the direct and indirect parental time costs of children in France and Italy, two countries that, in spite of some common traits, have very different family contexts. France is characterized by replacement-level fertility, generous family–friendly policies, more balanced gender roles and more relaxed norms on domestic standards, while Italy is marked by persistent low fertility as well as limited and sometimes inconsistent family policies along with more conservative values and attitudes.

The analyses were carried out using recent time-use surveys available in Italy (2008–2009) and France (2009–2010). Time budgets represent a unique source of information for this purpose, even if their cross-sectional design does not allow us to measure the causal effect of having children. Despite these limitations, we show that different allocations of parental time are associated with the presence of children, their number and age. Children are particularly time intensive in both countries, especially for women. The *direct time cost of children* (i.e. the time parents devote to childcare as a primary activity) is higher with several children, with clear decreasing marginal costs, and it decreases substantially as the age of the youngest child increases. The direct cost of children is always higher in Italy than in France (H1), whatever the number and age of children. However, the greatest differences between France and Italy concern families with at least one young child.

Faced with this huge increase in childcare time, Italian women adjust by substituting housework—which remains higher than among the French peers—with childcare and commonly try to preserve their free time by adjusting their labour supply. These larger adjustments in Italy may be explained by the lower level of childcare provision and the greater difficulty in outsourcing housework, the longer hours of working time and the stronger normative pressure on women to stop working in order to care for their young children. Unfortunately, our models do not allow us to determine which determinant prevails in explaining these cross-country differences. However, a combination of different factors is very likely. It is plausible that norms and values play a role: for instance, the idea that the family provides higher-quality childcare than external services, or that the preschool child suffers when the mother goes out to work. Nor can we disregard other structural constraints such as the lack of good and affordable alternative services in the market sector for outsourcing care tasks. However, the fact that almost every child in Italy aged 3–5 attends nursery school is a sign that the Italians are not so adverse to delegating childcare, but the scarcity of day-care centres and their high cost could make it particularly difficult when children are younger.

Our results highlight that the gender imbalance in childcare is surprisingly similar in Italy and France, as mothers perform around 65% of the childcare in both countries. Moreover, in absolute terms, Italian men seem to spend more hours caring for their children than their French peers. Conversely, in Italy a much more pronounced gender gap is observed in time spent on domestic work than in France. The hypothesis (H2) seems to be confirmed, whereby more balanced gender roles and lower normative constraints regarding housework mean that French women have lower time costs than their Italian peers in terms of additional housework.

The previous literature has shown that an individual’s free time has become more and more valuable in modern societies and that it competes with other pursuits such as family and working life. Our results show that the loss of free time is more pronounced for Italian parents, especially when they have preschool children, despite the fact that in Italy the amount of free time is also substantially lower among couples without children. The hypothesis whereby the reduction in free time for parents in Italy might be counterbalanced by lower labour market participation (H3) compared to France is therefore not confirmed. The total daily workload is much greater in Italy than in France.

The loss of free time is always more marked for women than for men in both countries. The two spouses specialize in paid and unpaid work, but this specialization is still much more pronounced in Italy than in France (H4). In France, women’s free time is only partially affected by the number of children, contrary to Italy. Less free time for women also means that their average daily workload (both paid and unpaid work) is still higher than that of men, a result which contrasts with some previous findings that show a gender convergence of total working time (Burda et al. 2013). Furthermore, if we consider that part of this working time is partially unpaid for women, the time cost is only the tip of the iceberg regarding gender inequality. From a gender perspective, this article also underlines that there is a difference in both the quantity and the nature of partners’ child costs. The forgone leisure approach partially conceals the fact that part of the child cost for women also takes the form of fewer individual monetary resources due to shorter working hours or interrupted labour market participation. This marital specialization might appear to be efficient for couples with children in the short term, but it may turn out to be the wrong choice for women (as well as for the children’s well-being) in the event of union disruption or even in the long-term perspective of the mother’s reduced accumulation of pension rights. In this light, the investment in domestic work may be seen as a risky marital investment for women and as an additional long-term cost of children.

Paradoxically, we can envisage a sort of trap for Italian women: high domestic standards and related social expectations might prevent Italian women not only from having paid work but also from having children (or more children). In places like Italy, where the cost of children is disproportionately high and the compression of free time is remarkable, policies might play a mitigating role in keeping childrearing compatible with a reasonable quantity of free time. Could it be that Italian couples refrain from having more children due to the high time cost of children in Italy—especially the direct time cost? This is a difficult question to answer, since the causality might also be reversed. If the normative pressure for maintaining high childcare and domestic standards is perceived by Italian women to be so important, very low fertility might be seen as a strategy to enjoy maternity without substantially reducing the family’s domestic comfort. Parents may reduce fertility to increase their well-being and happiness (Billari 2009). This is particularly true for parents living in a country, such as Italy, where work and childbearing are difficult to reconcile and where the compression of free time is substantial for parents. What is certain is that social support and dedicated policies in Italy—especially for children under 3 years old—could be helpful in reducing the time cost of children for parents as well as the “gendered” cost for women in this delicate phase.

## Appendix

See Tables 5, 6, 7, 8, 9, 10 and Fig. 7.

**Table 5:** Fertility levels in France and Italy (cohort 1965)

Country	Parity				Completed fertility rates
	No child	1 child	2 children	3 + children	
France	10.3	18.4	41.2	30.2	2.0
Italy	18	25	40	17	1.5

*Source*: for France OECD (2011), for Italy, Istat, Tavole di fecondità regionali—estimated data

**Table 6:** Sample description (%)

	Italy			France		
	Total	Childless	With children	Total	Childless	With children
*Family composition*						
Childless	0.21	1.00	0.00	0.31	1.00	0.00
1 child, 0–2	0.10	0.00	0.13	0.10	0.00	0.14
1 child, 3–5	0.08	0.00	0.10	0.06	0.00	0.09
1 child, 6–12	0.10	0.00	0.13	0.05	0.00	0.08
2 children, the youngest 0–2	0.10	0.00	0.13	0.10	0.00	0.14
2 children, the youngest 3–5	0.10	0.00	0.12	0.09	0.00	0.13
2 children, the youngest 6–12	0.20	0.00	0.25	0.13	0.00	0.19
3 children, the youngest 0–2	0.03	0.00	0.04	0.05	0.00	0.07
3 children, the youngest 3–5	0.03	0.00	0.03	0.05	0.00	0.07
3 children, the youngest 6–12	0.05	0.00	0.07	0.07	0.00	0.09
*Women age group*						
F age 20–34	0.33	0.44	0.30	0.42	0.50	0.39
F age 35–44	0.53	0.35	0.58	0.41	0.15	0.53
F age 45–54	0.14	0.22	0.12	0.17	0.35	0.09
*Men age group*						
M age 20–34	0.17	0.29	0.14	0.33	0.44	0.28
M age 35–44	0.55	0.41	0.59	0.43	0.19	0.54
M age 45–54	0.27	0.30	0.27	0.24	0.37	0.18
*Education level*						
Both high education	0.09	0.09	0.08	0.07	0.08	0.06
Both middle education	0.21	0.23	0.21	0.23	0.24	0.22
Both low education	0.34	0.29	0.35	0.33	0.30	0.34
M high, F lower	0.05	0.04	0.05	0.10	0.10	0.09
M middle, F high	0.06	0.08	0.06	0.06	0.07	0.06
M middle, F low	0.09	0.08	0.09	0.14	0.11	0.15
M low, F higher	0.17	0.18	0.16	0.09	0.10	0.08
*Labour supply*						
Both full time	0.39	0.55	0.34	0.54	0.63	0.51
M full time, F part time	0.18	0.13	0.20	0.11	0.07	0.13
M full time, F housewife	0.35	0.23	0.39	0.18	0.11	0.21
M no full time	0.08	0.08	0.07	0.16	0.19	0.15
*Marital status*						
Married couples	0.90	0.79	0.93	0.61	0.46	0.68
Cohabiting couples	0.10	0.21	0.07	0.39	0.54	0.32
*Self*-*perceived economic resources*						
Economic resources—fine	0.02	0.02	0.02			
Economic resources—adequate	0.61	0.64	0.60			
Economic resources—scarce	0.37	0.34	0.38			
*Income quartile*						
Less than q1				0.15	0.09	0.18
Between q1 and q2				0.23	0.16	0.27
Between q2 and q3				0.30	0.30	0.30
More than q3				0.31	0.45	0.25
Paid domestic aid	0.06	0.03	0.07	0.09	0.05	0.11
*Municipality size*						
City (metropolitan area or suburbs)	0.20	0.22	0.19	0.43	0.45	0.42
Medium-sized town (> 50.000 inhabitants)	0.43	0.40	0.43	0.19	0.19	0.19
Small town (< 50.000 inhabitants)	0.37	0.38	0.37	0.38	0.35	0.40
*Geographical area*						
Northern Italy	0.47	0.54	0.46			
Central Italy	0.16	0.15	0.16			
Southern Italy	0.37	0.31	0.39			
*Geographical area*						
Parisian area				0.17	0.17	0.16
Outside Parisian area				0.83	0.83	0.84
*Day of the week*						
Weekday	0.37	0.35	0.38	0.58	0.57	0.58
Sunday	0.31	0.33	0.30	0.22	0.23	0.21
Saturday	0.32	0.32	0.32	0.20	0.20	0.21
*N*	3597	768	2829	3781	1161	2620

**Table 7:** Mean value of childcare and housework according to different definitions in Italy and France (hours per week)

	Italy						France					
	Total		Parents		Childless people		Total		Parents		Childless people	
	Mean	SD	Mean	SD	Mean	SD	Mean	SD	Mean	SD	Mean	SD
Childcare primary activity												
Women	1.6	1.9	2.1	1.9	0	0	1.3	1.7	1.9	1.8	0.1	0.5
Men	0.8	1.2	1.0	1.3	0	0	0.7	1.1	0.9	1.3	0.1	0.4
Couple	2.4	2.6	3.0	2.6	0	0	2.0	2.4	2.8	2.4	0.2	0.7
Childcare secondary activity												
Women	0.5	1.0	0.6	1.1	0	0	0.3	0.9	0.4	1.1	0	0.4
Men	0.2	0.5	0.2	0.6	0	0	0.1	0.5	0.1	0.6	0	0.2
Couple	0.7	1.3	0.9	1.4	0	0	0.4	1.1	0.6	1.3	0.1	0.5
Time in the presence of children												
Women	5.2	4.0	6.5	3.4	0.2	0.6	5.1	4.9	7.1	4.3	0.6	2.1
Men	3.3	3.1	4.1	3.0	0.3	0.8	3.6	4.1	5.0	4.0	0.4	1.9
Couple	8.5	6.3	10.7	5.2	0.5	1.2	8.7	8.1	12.1	7.1	1.0	3.8
Housework primary activity												
Women	4.8	2.7	5.0	2.6	4.3	2.7	3.1	2.2	3.2	2.1	2.9	2.3
Men	1.6	2.0	1.5	2.0	1.9	2.2	2.0	2.2	2.0	2.2	2.0	2.2
Couple	6.5	3.6	6.5	3.5	6.3	4.0	5.1	3.5	5.2	3.4	4.9	3.7
Housework secondary activity												
Women	0.1	0.3	0.1	0.3	0	0.3	0.2	0.7	0.2	0.6	0.2	0.7
Men	0.0	0.2	0	0.1	0	0.2	0.1	0.5	0.1	0.5	0.1	0.4
Couple	0.1	0.4	0.1	0.4	0	0.3	0.3	0.9	0.3	0.9	0.3	0.8
*N*	3597		2829		768		3781		2620		1161	

*Source*: Italian Time-Use Survey 2008–2009 and French Time-Use Survey 2009–2010; authors’ computations

**Table 8:** Results for couples in Italy and France

Variables	Italy									France								
	Childcare			Housework			Free time			Childcare			Housework			Free time		
	Coeff.		S.E.	Coeff.		S.E.	Coeff.		S.E.	Coeff.		S.E.	Coeff.		S.E.	Coeff.		S.E.
Intercept	0.270	**	0.134	5.194	***	0.228	29.102	***	0.349	− 0.096		0.163	3.998	***	0.304	30.129	***	0.483
Couple typology (childless)																		
1 child, 0–2	4.466	***	0.127	− 0.687	***	0.217	− 3.278	***	0.331	3.776	***	0.111	− 0.144		0.206	− 3.120	***	0.328
1 child, 3–5	2.978	***	0.139	− 0.491	**	0.237	− 2.098	***	0.362	2.144	***	0.133	0.239		0.247	− 2.029	***	0.392
1 child, 6–12	1.739	***	0.128	− 0.168		0.218	− 2.032	***	0.334	1.340	***	0.138	0.109		0.256	− 1.623	***	0.407
2 children, the youngest 0–2	5.157	***	0.128	− 0.427	**	0.217	− 4.155	***	0.332	4.343	***	0.115	0.469	**	0.214	− 3.849	***	0.340
2 children, the youngest 3–5	3.238	***	0.132	0.106		0.224	− 3.287	***	0.343	2.607	***	0.119	0.117		0.221	− 2.466	***	0.352
2 children, the youngest 6–12	1.798	***	0.110	0.333	**	0.186	− 2.357	***	0.285	1.574	***	0.108	0.350	*	0.201	− 1.391	***	0.320
3 children, the youngest 0–2	4.587	***	0.196	0.113		0.334	− 4.420	***	0.510	4.427	***	0.153	0.732	***	0.285	− 4.285	***	0.453
3 children, the youngest 3–5	3.336	***	0.215	0.335		0.365	− 4.294	***	0.558	2.625	***	0.153	0.229		0.285	− 2.243	***	0.452
3 children, the youngest 6–12	1.590	***	0.168	0.679	**	0.285	− 2.624	***	0.436	1.669	***	0.136	0.706	***	0.253	− 2.006	***	0.403
Women age group (ref = 35–44)																		
F age 20–34	0.012		0.090	− 0.361	**	0.153	− 0.125		0.233	0.060		0.087	− 0.549	***	0.162	0.558	**	0.257
F age 45–54	− 0.005		0.115	0.752	***	0.195	− 0.030		0.298	0.178	*	0.108	0.552	***	0.201	− 0.527	*	0.319
Men age group (ref = 35–44)																		
M age 20–34	− 0.064		0.104	− 0.132		0.177	0.013		0.271	− 0.092		0.088	− 0.322	**	0.163	0.359		0.259
M age 45–54	− 0.222	***	0.092	0.076		0.156	− 0.130		0.239	− 0.051		0.092	0.246		0.171	0.120		0.272
Education level (ref = both middle education)																		
Both high education	0.314	**	0.135	− 0.442	**	0.229	0.537		0.350	− 0.113		0.132	0.065		0.245	1.414	***	0.389
Both low education	− 0.359	***	0.094	0.100		0.160	− 0.474	**	0.245	0.184	**	0.088	− 0.123		0.164	0.270		0.261
M high, F lower	0.044		0.164	− 0.635	**	0.279	0.655		0.427	− 0.094		0.110	0.123		0.206	0.366		0.326
M middle, F high	0.188		0.149	− 0.315		0.252	0.188		0.386	− 0.153		0.133	− 0.082		0.247	− 0.394		0.393
M middle, F low	− 0.208		0.132	0.076		0.224	− 0.543		0.343	− 0.039		0.101	0.324	*	0.189	− 0.318		0.300
M low, F higher	− 0.007		0.107	− 0.172		0.182	− 0.327		0.278	0.208	*	0.115	− 0.126		0.215	0.361		0.341
Labour supply (ref = both full time)																		
M full time, F part time	0.154	*	0.094	0.316	**	0.158	0.901	***	0.242	0.094		0.095	0.413	**	0.176	0.292		0.280
M full time, F housewife	0.351	***	0.084	1.417	***	0.142	1.652	***	0.217	0.456	***	0.088	0.711	***	0.163	2.001	***	0.259
M no full time	0.549	***	0.137	1.834	***	0.231	2.165	***	0.353	0.378	***	0.086	0.745	***	0.160	2.418	***	0.254
Marital status (ref = married couples)																		
Cohabiting couples	0.201	*	0.115	− 0.645	***	0.195	0.357		0.298	0.173	***	0.066	− 0.082		0.122	0.243		0.194
Self-perceived economic resources (ref = adequate)																		
Economic resources—fine	− 0.111		0.263	0.055		0.445	− 0.134		0.681									
Economic resources—scarce	0.083		0.072	0.480	***	0.122	0.087		0.186									
Income quartile (ref = between q1 and q2)																		
Less than q1										− 0.289	***	0.100	0.117		0.187	0.096		0.296
Between q2 and q3										− 0.026		0.083	− 0.134		0.155	− 0.472	*	0.246
More than q3										0.016		0.093	− 0.116		0.172	− 0.624	**	0.274
Paid domestic aid (ref = no)																		
Yes	0.107		0.146	− 0.581	**	0.247	0.671	**	0.377	0.122		0.108	− 0.422	**	0.201	− 0.166		0.319
Municipality size (ref = medium-sized town)																		
City	0.050		0.089	0.051		0.151	− 0.312		0.231	0.144	*	0.083	− 0.174		0.154	0.222		0.244
Small town	− 0.104		0.074	0.157		0.126	− 0.207		0.193	− 0.048		0.081	0.333	**	0.150	− 0.297		0.238
Geographical area (ref = Northern Italy)																		
Central Italy	− 0.112		0.096	− 0.028		0.162	− 0.194		0.248									
Southern Italy	− 0.369	***	0.077	0.020		0.131	− 0.374	**	0.200									
Geographical area (ref = Parisian area)																		
Outside Parisian area										0.021		0.084	0.291	*	0.157	0.144		0.249
Day of the week (ref = week day)																		
Sunday	− 0.367	***	0.079	− 0.213		0.134	10.974	***	0.205	− 0.213	***	0.072	0.829	***	0.134	9.219	***	0.213
Saturday	− 0.157	**	0.079	1.947	***	0.133	4.728	***	0.204	− 0.223	***	0.073	2.207	***	0.136	6.408	***	0.217
*R* ^2^	0.449			0.238			0.469			0.450	***		0.127	***		0.423		
***N***	3597			3597			3597			3781			3781			3781		
Cross-equation correlations																		
Rho childcare/housework	− 0.039	**								0.018								
Rho childcare/leisure	− 0.252	***								− 0.221	***							
Rho housework/leisure	− 0.248	***								− 0.151	**

**Table 9:** Results for women and men in Italy

Variables	Italy																	
	Female									Male								
	Childcare			Housework			Free time			Childcare			Housework			Free time		
	Coeff.		SE	Coeff.		SE	Coeff.		SE	Coeff.		SE	Coeff.		SE	Coeff.		SE
Intercept	0.010		0.094	3.848	***	0.151	14.560	***	0.175	− 0.077		0.067	1.325	***	0.119	14.095	***	0.203
Family composition (ref = childless)																		
1 child, 0–2	3.003	***	0.095	− 0.432	***	0.152	− 1.926	***	0.178	1.457	***	0.069	− 0.381	***	0.122	− 1.333	***	0.211
1 child, 3–5	1.865	***	0.103	− 0.060		0.166	− 1.355	***	0.194	1.122	***	0.076	− 0.512	***	0.134	− 0.752	***	0.232
1 child, 6–12	1.174	***	0.095	0.147		0.152	− 1.283	***	0.179	0.575	***	0.070	− 0.273	**	0.124	− 0.796	***	0.213
2 children, the youngest 0–2	3.480	***	0.094	− 0.173		0.151	− 2.559	***	0.178	1.685	***	0.069	− 0.392	***	0.123	− 1.595	***	0.212
2 children, the youngest 3–5	2.136	***	0.097	0.348	**	0.156	− 2.099	***	0.183	1.097	***	0.071	− 0.316	***	0.126	− 1.236	***	0.218
2 children, the youngest 6–12	1.158	***	0.081	0.588	***	0.129	− 1.675	***	0.151	0.631	***	0.059	− 0.228	**	0.104	− 0.765	***	0.179
3 children, the youngest 0–2	2.969	***	0.145	0.312		0.234	− 2.789	***	0.274	1.605	***	0.107	− 0.274		0.190	− 1.664	***	0.327
3 children, the youngest 3–5	2.102	***	0.159	0.497	**	0.255	− 2.351	***	0.299	1.200	***	0.116	− 0.201		0.206	− 2.071	***	0.356
3 children, the youngest 6–12	1.090	***	0.123	0.781	***	0.198	− 2.139	***	0.232	0.490	***	0.091	0.023		0.161	− 0.591	**	0.278
Age group (35–44)																		
20–34	0.036		0.058	− 0.162	**	0.092	− 0.048		0.103	− 0.022		0.051	− 0.171	**	0.089	− 0.144		0.146
45–54	− 0.142	**	0.074	0.424	***	0.119	− 0.170		0.133	− 0.081	**	0.043	0.086		0.076	0.143		0.126
Education (ref = middle)																		
High education	0.426	***	0.072	− 0.419	***	0.116	0.389	***	0.130	0.108	**	0.057	0.166	**	0.101	0.334	**	0.166
Low education	0.186	***	0.055	− 0.166	**	0.087	0.297	***	0.097	0.156	***	0.039	0.137	**	0.069	0.160		0.113
Woman’s labour supply (ref = full time)																		
Part time	0.127	**	0.067	0.439	***	0.108	0.586	***	0.127	0.032		0.049	− 0.098		0.087	0.368	***	0.151
Not employed	0.479	***	0.060	1.808	***	0.097	1.316	***	0.113	− 0.126	***	0.043	− 0.338	***	0.077	0.429	***	0.133
Men’s labour supply (ref = full time)																		
Either part time or not employed	− 0.012		0.094	0.043		0.150	− 0.332	**	0.177	0.344	***	0.069	1.042	***	0.122	1.445	***	0.211
Marital status (ref = married couples)																		
Cohabiting couples	0.156	**	0.085	− 0.517	***	0.137	0.129		0.161	0.041		0.063	− 0.133		0.111	0.276		0.192
Self-perceived economic resources (ref = adequate)																		
Economic resources—fine	− 0.111		0.195	− 0.098		0.313	0.080		0.367	− 0.017		0.143	0.124		0.254	− 0.210		0.439
Economic resources—scarce	0.060		0.053	0.243	***	0.085	− 0.066		0.100	0.001		0.039	0.246	***	0.069	0.051		0.119
Paid domestic aid (ref = no)																		
Yes	0.071		0.107	− 0.433	***	0.173	0.509	***	0.202	0.062		0.079	− 0.149		0.140	0.265		0.241
Municipality size (ref = medium-sized town)																		
City	0.027		0.066	− 0.077		0.106	− 0.056		0.124	0.020		0.048	0.132		0.086	− 0.276	**	0.148
Small town	− 0.012		0.055	0.137		0.088	− 0.195	**	0.103	− 0.107	***	0.040	0.032		0.072	− 0.080		0.124
Geographical area (ref = Northern Italy)																		
Central Italy	− 0.049		0.071	0.070		0.114	− 0.295	**	0.134	− 0.065		0.052	− 0.119		0.093	0.113		0.160
Southern Italy	− 0.169	***	0.057	0.392	***	0.092	− 0.508	***	0.108	− 0.209	***	0.042	− 0.410	***	0.074	0.107		0.129
Day of the week (ref = week day)																		
Sunday	− 0.591	***	0.059	− 0.703	***	0.094	4.538	***	0.111	0.230	***	0.043	0.505	***	0.077	6.435	***	0.132
Saturday	− 0.337	***	0.058	0.568	***	0.093	1.749	***	0.110	0.179	***	0.043	1.369	***	0.076	2.963	***	0.131
*R* ^2^	0.419			0.237			0.378			0.240			0.126			0.418		
*N*	3597																	
Cross-equation correlations																		
Rho F childcare/F housework	− 0.045	***																
Rho F childcare/F free time	− 0.294	***																
Rho F childcare/H childcare	0.181	***																
Rho F childcare/H housework	− 0.072	***																
Rho F childcare/H free time	− 0.145	***																
Rho F housework/F free time	− 0.352	***																
Rho F housework/H childcare	− 0.010																	
Rho F housework/H housework	0.224	***																
Rho F housework/H free time	− 0.132	***																
Rho F free time/H childcare	− 0.113	***																
Rho F free time/H housework	− 0.095	***																
Rho F free time/H free time	0.407	***																
Rho H childcare/H housework	0.056	***																
Rho H childcare/H free time	− 0.093	***																
Rho H housework/H free time	− 0.081	***

**Table 10:** Results for women and men in France

Variables	France																	
	Female									Male								
	Childcare			Housework			Free time			Childcare			Housework			Free time		
	Coeff.		SE	Coeff.		SE	Coeff.		SE	Coeff.		SE	Coeff.		SE	Coeff.		S.E.
Intercept	0.000	***	0.000	2.524	***	0.178	15.543	***	0.252	− 0.228	**	0.088	1.410	***	0.184	14.742	***	0.278
Family composition (ref = childless)																		
1 child, 0–2	2.485	***	0.081	− 0.222	*	0.127	− 1.838	***	0.184	1.280	***	0.064	− 0.015		0.133	− 1.125	***	0.204
1 child, 3–5	1.371	***	0.096	0.124		0.152	− 1.283	***	0.220	0.779	***	0.076	0.112		0.159	− 0.727	***	0.243
1 child, 6–12	0.887	***	0.099	0.161		0.158	− 0.966	***	0.228	0.469	***	0.079	0.060		0.165	− 0.769	***	0.253
2 children, the youngest 0–2	2.807	***	0.083	0.297	**	0.132	− 2.446	***	0.191	1.521	***	0.065	0.108		0.137	− 1.359	***	0.209
2 children, the youngest 3–5	1.661	***	0.083	0.150		0.135	− 1.700	***	0.195	0.945	***	0.067	− 0.018		0.140	− 0.788	***	0.214
2 children, the youngest 6–12	1.026	***	0.073	0.261	**	0.123	− 0.976	***	0.177	0.547	***	0.060	0.194		0.125	− 0.517	***	0.191
3 children, the youngest 0–2	3.279	***	0.109	0.330	*	0.175	− 2.526	***	0.253	1.145	***	0.087	0.351	*	0.183	− 1.824	***	0.279
3 children, the youngest 3–5	1.682	***	0.108	0.386	**	0.175	− 1.442	***	0.252	0.947	***	0.087	− 0.142		0.182	− 0.851	***	0.278
3 children, the youngest 6–12	1.079	***	0.096	0.413	***	0.156	− 1.207	***	0.225	0.603	***	0.077	0.430	***	0.162	− 0.834	***	0.247
Age group (ref = 35–44)																		
20–34	− 0.048		0.052	− 0.379	***	0.083	0.239	**	0.115	0.033		0.043	− 0.248	***	0.090	0.112		0.132
45–54	0.009		0.067	0.484	***	0.108	− 0.202		0.150	0.057		0.046	0.104		0.095	0.017		0.139
Education (ref = middle)																		
High education	− 0.103		0.068	0.207	**	0.105	− 0.081		0.144	0.020		0.050	− 0.088		0.102	0.687	***	0.149
Low education	0.033		0.051	− 0.051		0.079	0.103		0.109	0.206	***	0.039	− 0.116		0.081	0.130		0.118
Woman’s labour supply (ref = full time)																		
Part time	0.110	*	0.063	0.147		0.097	0.330	**	0.140	− 0.017		0.049	0.088		0.102	− 0.390	**	0.156
Not employed	0.628	***	0.060	1.062	***	0.094	1.890	***	0.136	− 0.101	**	0.047	− 0.196	**	0.099	0.393	***	0.151
Men’s labour supply (ref = full time)																		
Either part time or not employed	− 0.028		0.060	− 0.142		0.093	0.107		0.135	0.237	***	0.047	0.608	***	0.099	1.650	***	0.151
Marital status (ref = married couples)																		
Cohabiting couples	0.125	***	0.047	− 0.150	**	0.076	0.214	*	0.109	0.055		0.038	0.017		0.079	0.194		0.121
Income quartile (ref = between q1 and q2)																		
Less than q1	− 0.206	***	0.076	0.229	*	0.118	− 0.202		0.170	− 0.140	**	0.059	− 0.311	**	0.124	0.015		0.189
Between q2 and q3	− 0.047		0.058	− 0.042		0.095	− 0.168		0.138	0.046		0.048	− 0.005		0.100	− 0.280	*	0.153
More than q3	0.038		0.062	− 0.022		0.104	− 0.258	*	0.149	0.016		0.052	0.001		0.109	− 0.373	**	0.167
Paid domestic aid (ref = no)																		
Yes	0.011		0.081	− 0.270	**	0.124	− 0.102		0.180	0.107	*	0.062	− 0.108		0.131	− 0.084		0.200
Municipality size (ref = medium-sized town)																		
City	0.072		0.054	− 0.081		0.095	0.123		0.137	0.104	**	0.048	− 0.092		0.101	0.139		0.153
Small town	− 0.049		0.057	0.155	*	0.093	− 0.024		0.135	0.041		0.047	0.206	**	0.098	− 0.242		0.150
Geographical area (ref = Parisian area)																		
Outside Parisian area	0.091	*	0.054	0.088		0.097	− 0.075		0.140	− 0.037		0.049	0.226	**	0.103	0.141		0.157
Day of the week (ref = week day)																		
Sunday	− 0.334	***	0.054	0.238	***	0.083	3.909	***	0.121	0.130	***	0.042	0.598	***	0.088	5.305	***	0.134
Saturday	− 0.353	***	0.054	0.906	***	0.085	2.803	***	0.123	0.140	***	0.043	1.306	***	0.089	3.617	***	0.137
*R* ^2^	0.640			0.128			0.327			0.211			0.088			0.371		
*N*	3781																	
Cross-equation correlations																		
Rho F childcare/F housework	0.052	***																
Rho F childcare/F free time	− 0.208	***																
Rho F childcare/H childcare	0.110	***																
Rho F childcare/H housework	− 0.027	***																
Rho F childcare/H free time	− 0.109	***																
Rho F housework/F free time	− 0.147	***																
Rho F housework/H childcare	− 0.041	***																
Rho F housework/H housework	0.226	***																
Rho F housework/H free time	− 0.086																	
Rho F free time/H childcare	− 0.124	***																
Rho F free time/H housework	− 0.083	*																
Rho F free time/H free time	0.381	***																
Rho H childcare/H housework	0.046	***																
Rho H childcare/H free time	− 0.121	***																
Rho H housework/H free time	− 0.102																	

## Footnotes

1 Tables 6 and 7 gives the mean value of childcare according to different definitions: active childcare, childcare as secondary activity and time spent in the presence of children. For parents, childcare is mainly a primary activity. Time spent in the presence of children (passive childcare) is much greater than childcare as a primary and secondary activity (active childcare).

2 However, they might still differ on unobserved ones.

3 The shapes of the curves of each country are similar, but the differences between countries are larger (especially for housework) since the different population activity structures are not taken into account. Results are available from authors upon request.

4 We added the estimated constant to the coefficient estimated for each family type. It thus represents time use on weekdays. If we were to change the reference to the weekend, the shape of the curve would not change but would be shifted upward. When the coefficient is not statistically significant, the predicted value is equal to the constant value.

5 The coefficients of the linear regression estimate the marginal effect at the means (for an average day). The estimated constant represents the time spent on a certain activity on weekdays, by childless couples, women (or men), aged 35–44, married, medium educated, with medium income, not using domestic help, living in a medium-sized town, etc.

6 Regressions (not shown but available upon request) on childcare time as a secondary activity and on time in the presence of children show a similar decreasing age effect for the couple, but of reduced magnitude, since children need less direct care when they grow up.

7 The only exception is parents having an only child older than 6, who lose more free time in France than in Italy.
